# Physical activity in early childhood: a five-year longitudinal analysis of patterns and correlates

**DOI:** 10.1186/s12966-022-01289-x

**Published:** 2022-04-20

**Authors:** Linnea Bergqvist-Norén, Emilia Hagman, Lijuan Xiu, Claude Marcus, Maria Hagströmer

**Affiliations:** 1 Department of Clinical Science, Intervention and Technology - Division of Pediatrics, Karolinska Institutet, Blickagången 6A, Stockholm, Huddinge 141 57 Sweden; 2 Department of Neurobiology Care Sciences and Society - Division of Physiotherapy, Karolinska Institutet, Alfred Nobels Allé 23, Stockholm, Huddinge 141 83 Sweden; 3 Academic Primary Health Care Centre, Region Stockholm, Stockholm, Sweden

**Keywords:** Accelerometer, Childhood obesity, Early STOPP, Toddler

## Abstract

**Background:**

Knowledge on longitudinal patterns and related factors of young children’s physical activity (PA) is still scarce. Therefore, the aim of this study was to examine patterns and changes of accelerometer-measured PA over time in two to six-year-old children. Furthermore, the aim was to investigate if parental PA, socioeconomic status, sex, weight status, and motor skills are related to child PA over time, using prospective cohort data from a clustered randomized controlled trial.

**Methods:**

One hundred and six children (52% girls) and their parents had PA measured yearly from age two to six with an Actigraph GT3X. The actigraph was worn on the non-dominant wrist for one week; anthropometric data and motor skills, as well as background information, was collected simultaneously. The outcome was counts per minute from the vector magnitude, and linear mixed-effect models were used to answer the research questions.

**Results:**

Among the children, accelerometer-measured PA increased on average by 11% per year from two years of age (mean 3170 cpm (3007-3334 95% CI)) onwards to six years of age (mean 4369 cpm (4207-4533 95% CI)). From three years of age, children were more active on weekdays than on weekend days. The rate of difference varied across low, medium, and highly active children (based on tertiles). No significant differences in weekdays/weekend PA among the lowest active children was found. Despite this, they were still significantly less active on weekend days than the most active children. Maternal, but not paternal PA was found to be significantly positively related to child PA over time, with a medium to large effect size. But no significant relationships were found between child PA and sex, weight status, or socioeconomic status.

**Conclusions:**

PA increased on average with 11% per year, similarly for boys and girls. From three years of age children were more active during weekdays than weekend days. These results indicate that child PA benefits from active stimulation by parents and care takers already from early ages. It is important to identify attributes of possible intervention designs for weekend days for families with young children as well as characterize the least active children.

**Trial registration:**

Early STOPP was prospectively registered in the clinical trials registry: clinicaltrials.gov, ID NCT01198847

**Supplementary Information:**

The online version contains supplementary material available at 10.1186/s12966-022-01289-x.

## Background

Being sufficiently physically active in childhood is correlated with better cardiometabolic health, muscular fitness, psychological well-being, and bone health [1, 2]. Interventional studies aiming at enhancing physical activity (PA) behavior among children have shown limited effects [3, 4]. Reasons for this are most likely multifactorial but may include: poor delivery, limited adherence, insufficient intervention intensity, already-active periods targeted, or that the intervention was not suitable for the specific population [3]. The reviews by Metcalf et al. [3] and Biddle et al. [4] both concluded that much is still to be learned about child PA origins and related factors [3, 4]. Knowledge that is needed to be able to improve behavior change programs [4]. There is an urge to increase child PA, since a large number of children are insufficiently active [5]. The specific number of insufficiently active children differs across age groups and across countries; however, roughly 60% of children (2-9.9 years) and adolescents (>10 years) in Europe are insufficiently physically active [5].

Previously, age and sex has been related to children’s PA, where PA decreases yearly after the age of five to six years [6–8], and boys engage in more PA than girls [6, 9–11]. Also, a more well-developed motor function has been positively related to PA in preschool-aged children, however, the causality is unclear [12]. Children with normal weight seem to be on average less sedentary and more physically active than children with overweight or obesity, but results are inconclusive [6, 9, 13–17]. For children under the age of six, this association is rarely investigated [6]. Socioeconomic status (SES) has also been related to child PA with inconsistent results. Some have found children from high SES to be more physically active and less sedentary [6, 9, 18–21], while others have observed the opposite or no significant relation at all [6, 9, 22–25]. It is noteworthy that most of these studies focused on school-age children while children younger than six years are rarely included.

A relationship between parental and child PA has been studied with inconsistent results, where most studies were based on cross-sectional study designs, including school-aged children [26–38]. Parental PA correlated to child PA regardless of sex in some studies [26–31], while others have found correlations between either mothers’ or fathers’ PA to either boys’ or girls’ PA, or no significant correlations at all [33–39] regardless of age. The effect sizes and assessment methods (self-reported and/or sensor-based) of these studies have varied, and there is a lack of prospective study designs investigating parental-child PA relationships over time.

It has been shown that daily patterns of young children’s PA have an uneven distribution throughout the day and across the week [25, 36, 40, 41]. Three-year-old children were more active on weekdays than weekend days and in two-year-old Swedish children PA differed across the day [25, 36]. How the daily and weekly PA patterns change over time in young children is, to the best of our knowledge, unknown.

During recent decades, young children’s PA has received a great amount of attention [2] and therefore knowledge is increasing. However, for children under the age of six years, research is still scarce and patterns and correlates of PA still needs to be explored and determined, preferably using objective methods and prospective study designs, over a longer period, since this has been called for [2, 6].

In the present study, we aimed to determine patterns of accelerometer-measured PA and their changes over time, in young children from two to six years of age. Furthermore, the aim was to investigate if parental PA, socioeconomic status, sex, weight status, and motor skills are related to child PA over time.

## Methods

### Design and study population

Longitudinal prospective cohort data from a clustered randomized controlled trial, the Early STockholm Obesity Prevention Project (Early STOPP), was used. Early STOPP followed 238 children from one to six years of age. The project was initiated in 2009, recruitment started in 2010 and the project ended in June 2018. The children were recruited from child healthcare centers in the Stockholm region and were included based on obesity risk, estimated from parental body mass index (BMI). High risk (*N *= 181) was defined as having two parents with overweight (BMI 25-29.9 kg/m^2^) or at least one parent with obesity (BMI ≥30 kg/m^2^), and low risk (*N *= 57) was defined as having both parents with normal weight (BMI <25 kg/m^2^). The high-risk group children were randomized to either intervention (*N *= 66) or control (*N *= 115), based on the cluster (the child healthcare centers) and the low-risk children were included as a reference group. Detailed information about the project can be found in the study protocol [42]. In the present study, the intervention group was excluded due to the possible intervention effect. Briefly, the intervention was a low intensive coaching intervention with the focus on child physical activity, sleep, and dietary habits. The control group and the reference group did not receive any intervention or additional information other than the aims and scope of the study. In total 172 children (52.1% girls) were eligible for inclusion. A flowchart of sample attrition is presented in Aditional file 1.

Early STOPP was approved by the Stockholm Regional Ethics Committee in Stockholm in March 2009 (file no. 2009/217-31), and the parents signed a written consent to be a part of the study.

### Physical activity

PA was measured yearly between two to six years of age using a tri-axial accelerometer Actigraph GT3X (Actigraph, Pensacola, FL), and was analyzed in the ActiLife program, version 6.11.9. (Actigraph, Pensacola, FL). A sampling rate of 30 hz was applied. We used the outcome variable counts per minute (CPM) from the vector magnitude (VM) that combines CPM from three axes into one outcome defined as √(x^2^+y^2^+z^2^). The VM has a higher correlation to total physical activity (energy expenditure) than the vertical axis alone [43]. We used data from the VM CPM to detect changes in total PA over time. There are no established thresholds for intensity levels developed for children during this broad time span. Movement ability changes during these years and one set of thresholds would not be suitable [44–46]. Nor would it be possible to evaluate the activity over time if different sets of thresholds were adopted [47]. Therefore, we used total PA as recommended by Karas et al [48].

Children and their parents wore the accelerometer on their non-dominant wrist for seven consecutive days. To be included in the present study, individuals had to provide activity data on at least four days, including one weekend day [49]. To ensure validity, a day had to include 10 hours of measurement with a minimum of 100 VM CPM [36, 50, 51]. Furthermore, for all years, nighttime sleep was removed for children between the hours of 21.00-07.00 and for parents between 23.00-06.00 after double-checking sleep diaries and accelerometer data. To be included in this study and the longitudinal analyses, an individual had to have PA data for at least two of the annual measurements [52].

### Weight status

Height was measured with a fixed stadiometer (Ulmer; Buss Design Engineering, Elchinge, Germany) to the nearest 0.1 cm, and body weight was measured with a portable scale Tanita HD-316 (Tanita Corp, Tokyo, Japan) to the nearest 0.1 kg. Both were professionally calibrated yearly.

Parental weight status was calculated using BMI; weight (kg) divided by height (m) squared. To classify adults as overweight or obese, standard cut-offs provided by the World Health Organization (WHO) were used and are as follows: overweight was defined as BMI 25-29.9 kg/m^2^ and obesity as BMI ≥30 kg/m^2^.

Child weight status was calculated using Body Mass Index Standard Deviation Score (BMI SDS sometimes referred to as BMI Z-score), an age and sex adjusted variable. We used the international reference provided by the International Obesity Task Force (IOTF) to classify overweight and obesity [53]. All calculations and classifications were performed yearly for parents and children.

### Motor skills

At age six, the Movement Assessment Battery for Children 2^nd^ edition (MABC-2) was used to assess motor skills. This test was designed for children aged three to 16 and we used the specific age range “one” for children between three to six years of age. MABC-2 consists of 11 parts that are scored separately, age adjusted, and then combined into one outcome. A dichotomous variable was created and children who scored at or below the 15^th^ percentile were classified as low [54]. This test was administered by a trained and qualified physical educational teacher. More information about the test can be found elsewhere [54].

### Demographics and family-related variables

In the present study, highest attained educational level was used to determine SES. Information on education was derived from questionnaires conducted at baseline or at the fifth wave follow up, in case of missing data at baseline. Parents reported completed educational level as either nine years, 12 years, or academic education. Family education was considered high if at least one parent had an academic education.

The following factors were included as described to be able to adjust for possible confounding effects. Daycare was reported yearly as in-home care or preschool care/similar, preschool care was defined as either full time ≥30 hours per week or part time <30 hours per week. Country of origin was assessed at baseline and was divided into Nordic or non-Nordic, where non-Nordic was defined as having at least one parent originating from a non-Nordic country. Current season for PA measurement was categorized as spring (March-May), summer (June-August), fall (September-November), and winter (December-February).

### Statistical analyses

Descriptive data were, unless specified otherwise, presented as mean and standard deviation or as frequencies (%). For group comparisons, independent samples *t*-tests and X^2^ tests were conducted as appropriate. To test the differences between weekdays and weekend days, paired t-tests were conducted for all years separately. Tertiles of child’s total PA were derived per year and defined as low, medium, or high activity. To analyze differences between weekdays and weekend days within the tertiles, paired t-tests were performed for each tertile for all years. To analyze differences between weekdays and weekend days between the tertiles, univariate analyses of variance were performed for each year with mean change between weekdays and weekend days as the outcome variable.

For the longitudinal analysis, linear mixed models (LMM) were used. As mentioned, to be included in the analyses a minimum of two valid PA-periods was set as a criterion; however, missing data were still common over the years. LMM is a flexible tool for analysis of repeated measures, with the advantage of dealing with missing values [55]. We used intercept only models, with the intercept as the only random effect. The overall change in child PA across ages was explored, adjusted for time. To explore relating factors to child PA, bivariate models were performed on all factors, including child PA over time, and then the factor of interest. Furthermore, two different multivariate models were then fitted using the Akaike information criterion as a model fit criterion. Child total PA was included as the dependent variable, nested to each individual code. Maternal PA, paternal PA, sex, risk-group, parental education, motor skills, ethnicity, season, and time in preschool care were included as fixed factors; BMISDS was included as a time varying variable in further analysis. Model I was a full model and included all factors. Model II was a forward stepwise selection model and included all factors with a *p*-value of <0.3 from bivariate models (additional file 8), adjusted for season and time in preschool, since these factors were significant in multivariate model I. Additional analyses were then made to further investigate the relating factors. Season of year was analyzed together with maternal PA and child time in preschool to investigate if these factors had any interaction.

Relative effect sizes for significant factors were expressed using Cohen’s *f*^2^ [56], suitable within the context of a multivariate regression model [57]. Interpretation for effect size using Cohen’s *f*^2^ are: small 0.02, medium 0.15, and large 0.35 [56].

All tests were two-sided, and significance level was set to 0.05. In cases of multiple testing, the Bonferroni correction was used.

All statistical analyses were performed using IBM SPSS Statistics for Windows, Version 26.0. (Armonk, NY: IBM Corp) and STATA StataCorp, 2019. Stata Statistical Software: Release 16. (College Station, TX: StataCorp LLC).

## Results

### Background – inclusion and baseline characteristics

Of the 172 eligible families, 106 had a minimum of two child accelerometer-measured PA periods and were included in the study (additional file 1). Of the included children, 17 had valid measures at all time points and 85% had three or more valid periods. Number of valid days per measure period were high across all years for children (all years mean 6.74 days) and parents (all years mean 6.84 days). The majority (95%) of the children had PA measured within one month from the child’s birthday. Families who did not fulfil criteria for inclusion (*N* = 66) were more likely to be in the high-risk group (*p<0.010)* and had a lower educational level (*p=0.035)* than included families. Baseline characteristics of included participants are provided in Table 1.

**Table 1:** Baseline characteristics of participants (*n *= 106)

	Mean (SD) or *n* (%)	
**Child**		
Risk group		
Low risk	43	(40.6)
Sex		
Girls	55	(51.9)
Age	2.04	(0.07)
BMI SDS	-0.08	(1.08)
**Mother**		
Age	34.8	(4.2)
BMI	27.6	(6)
Educational level^a^		
High	75	(70.8)
**Father**		
Age	37	(4.5)
BMI	26.7	(4.6)
Educational level		
High	59	(55.7)
**Family**		
Educational level^b^		
High	82	(77.4)
Ethnicity^c^		
Nordic	82	(77.4)

^a^ Educational level was considered high if an academic education was attained

^b^ Family educational level was considered high if at least one parent had attained academic education

^c^ Nordic ethnicity was considered if both parents had a Nordic country background

### PA Patterns

**Fig. 1:**
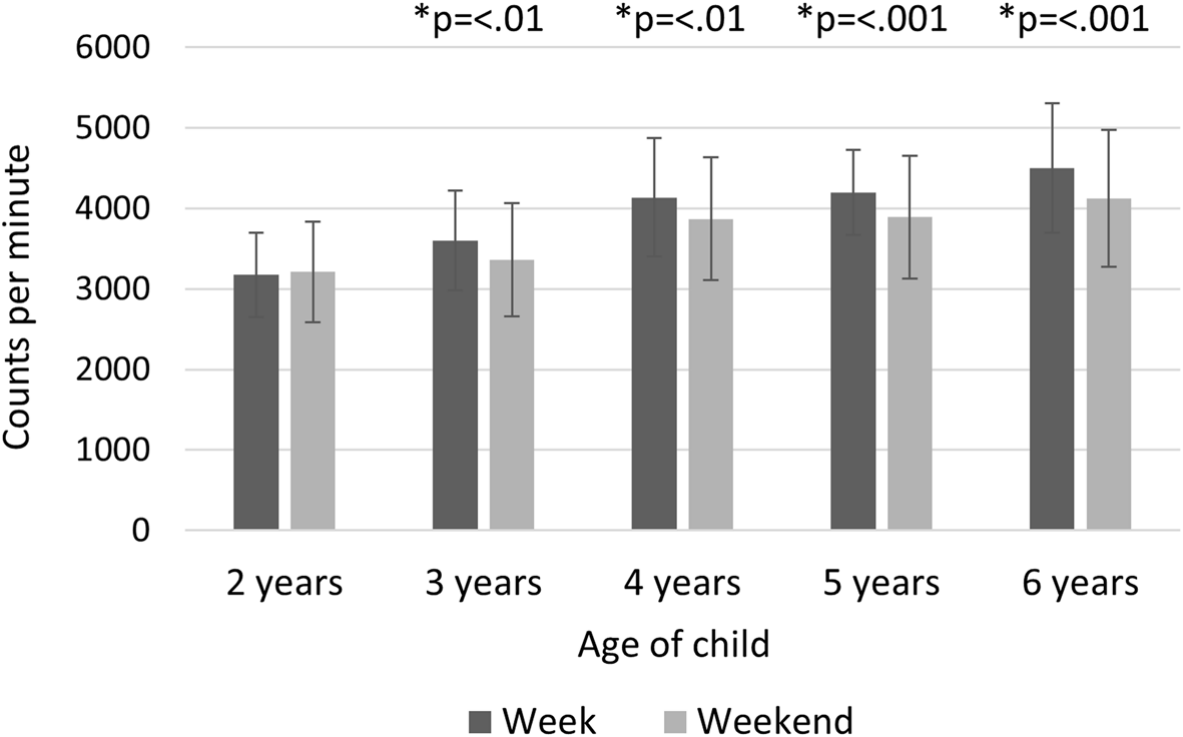
Child physical activity – weekdays and weekend days for all years. *p-value significant for differences between weekdays and weekend days. Error bars represent standard deviation

**Fig. 2:**
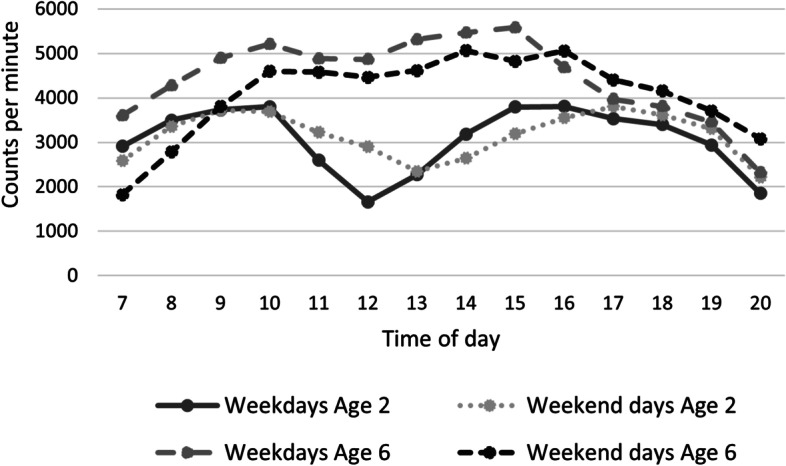
Hourly pattern of child physical activity. Comparison of hourly pattern of child physical activity on weekdays and weekend days between child aged two and six

Child accelerometer-measured PA increased on average by 11% per year from a mean of 3170 CPM (3007-3334 95% CI) at two years of age to a mean of 4369 CPM (4207-4533 95% CI) at six years of age (Fig. 1). This increase was observed across the whole day, even during “peak hours” (Fig. 2). The peak hours for PA were around mid-morning (approx. 10 am) and mid-afternoon (approx. 3 pm) across all years (Fig. 2  and Additional file 2). When studying differences between all the weekdays, no significant differences were observed (Additional file 3).

From three years of age, there were significant differences between weekdays and weekend days, with children being more active during weekdays (Fig. 1 and Additional file 4). These differences differed between children with low, medium, and high activity. Children with the highest activity differed significantly more than children with the lowest activity (Table 2).

**Table 2:** Differences in physical activity (vector magnitude counts per minute) between weekdays and weekend days by tertiles

Year	Activity group^a^	N	Mean PA weekday (SD^b^)	Mean PA weekend days (SD)	Mean difference^c^ (SD)	*p*-value for model^d^
2	Low	28	2621 (231)	2723 (494)	102 (490)	0.397
	Medium	29	3142 (147)	3105 (324)	-37 (310)	
	High	29	3744 (330)	3786 (498)	42 (341)	
3	Low	18	2970 (223)	3072 (485)	102 (475)	0.032*
	Medium	19	3517 (217)	3171 (647)	-346 (585)	
	High	19	4286 (422)	3835 (712)	-451 (841)	
4	Low	23	3396 (356)	3405 (698)	9 (787)	0.033*
	Medium	24	4064 (134)	3790 (509)	-273 (479)	
	High	23	4954 (512)	4418 (718)	-536 (771)	
5	Low	23	3640 (338)	3403 (717)	-236 (641)	0.163
	Medium	24	4221 (160)	4026 (664)	-195 (638)	
	High	23	4760 (284)	4244 (695)	-515 (560)	
6	Low	29	3697 (450)	3710 (898)	12 (784)	<0.001*
	Medium	29	4453 (184)	4077 (663)	-376 (674)	
	High	29	5360 (564)	4589 (746)	-771 (691)	

*p-value significant at a 0.05 level

^a^ Activity group based on tertiles

^b^ SD = Standard deviation

^c^ Crude mean difference between weekend days and weekdays

^d^ *p*-value for the full model. Adjustment for multiple comparison using Bonferroni reveals that:at year 3 low vs. high, *p*=0.039. Non-significant interaction between tertile low and medium or tertile medium and high at year 4 low vs. high, p=0.028. Non-significant interaction between tertile low and medium or tertile medium and high at year 6 low vs. high, *p*<0.001. Non-significant interaction between tertile low and medium or tertile medium and high

When analyzed separately, children in the lowest tertile did not differ in activity between weekdays and weekend days in any of the measured years (Table 3). Meanwhile, the highest tertile differed between weekdays and weekend days at all years, as did the middle tertile (Table 3). Even though activity levels of children with high activity differed more between weekdays and weekend days, these children were still significantly more active than the least active group (Additional file 5). At the age of two to five, season did not differ significantly between the activity tertiles. However, at age six significantly more children in the highest tertile were measured during summertime, therefore, at age six the analyses were further adjusted for season with similar results (model *p*-value 0.002). These described patterns did not differ between sexes at any measured year.

**Table 3:** Differences in physical activity between weekdays and weekend days by tertiles based on activity

Year	Tertile low				Tertile medium				Tertile high			
	N	WE^a^	WD^b^	*p*-value	N	WE	WD	p-value	N	WE	WD	*p*-value
Age 2	28	2621	2723	0.279	29	3142	3105	0.524	29	3744	3786	0.507
Age 3	18	2970	3072	0.375	19	3517	3171	0.019*	19	4286	3835	0.031*
Age 4	23	3396	3404	0.959	24	4064	3790	0.01*	23	4954	4418	0.003*
Age 5	23	3640	3403	0.091	24	4221	4026	0.147	23	4760	4244	<0.001*
Age 6	29	3697	3710	0.932	29	4453	4077	0.006*	29	5360	4589	<0.001*

* *p*-value significant at 0.05 level

^a^ Weekdays

^b^ Weekend days

### Parental accelerometer-measured physical activity over time

**Fig. 3:**
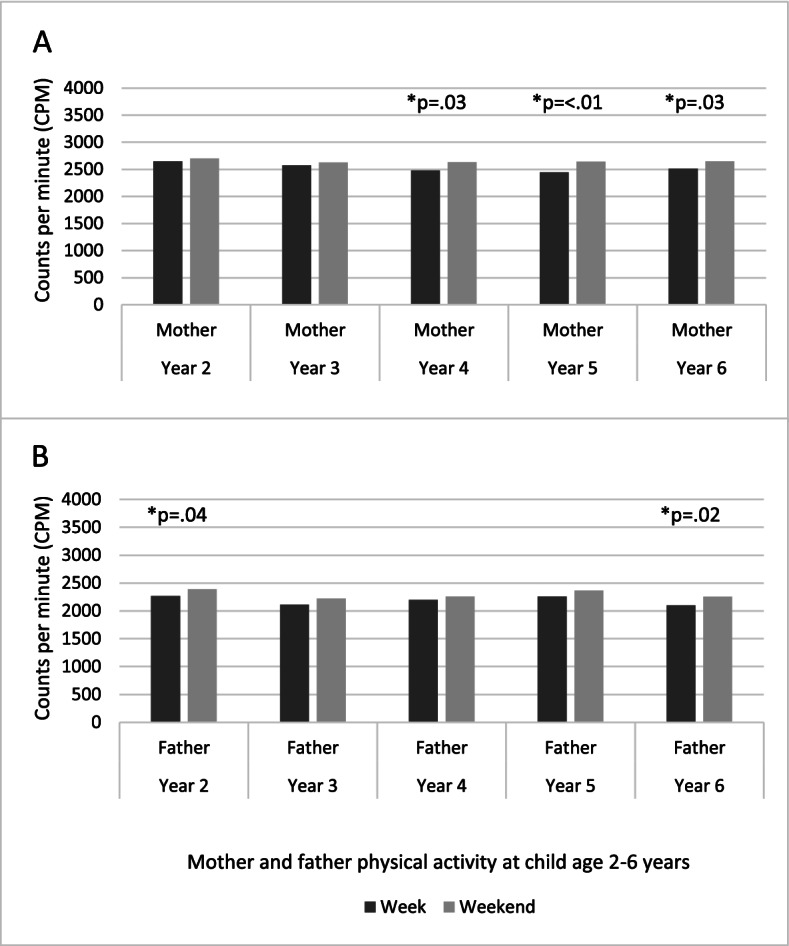
Parental physical activity – weekdays and weekend days for all years. *p-value significant for differences between weekdays and weekend days. Error bars represents standard deviations

For parents, PA did not differ significantly between the observed years. Across all measured years, mothers had a mean of 2570 (502 SD) CPM and fathers a mean of 2223 (526 SD) CPM (Fig. 3 and Additional file 4). Means and standard deviations for all years separately for parents and children are presented in Additional file 4. Mothers were significantly more physically active than fathers at all timepoints (Additional file 6). For mothers at child aged four, five and six and for fathers at child aged two and six, PA differed significantly between weekdays and weekend days, with both parents being more active on weekend days than weekdays (Fig. 3 and Additional file 4). For parents, peak hours for activity differs between weekdays and weekend days. During weekdays activity-peak occurs around 5 pm, while during weekend days the activity peaks around lunchtime (11 am-12 pm) (Additional file 7).

### Correlations to child accelerometer-measured PA over time

In mixed model I and II, maternal accelerometer-measured PA was significantly related to child accelerometer-measured PA over time (Table 4). Maternal PA was significantly related with a coefficient of 0.16. For every 1 maternal CPM, child PA was 0.16 CPM higher; this with an effect size of 0.32, medium effect based on Cohen’s *f*^2^. In bivariate models, parental education was significantly related with child PA (Additional file 8), but in none of the multivariate models did this correlation remain significant.

**Table 4:** Linear mixed effect multivariate models I and II

Multivariate model I					Multivariate model II			
	Coef.^h^	95% CI^i^		*p*-value	Coef.	95% CI		*p*-value
Sex	55.0	-143.7 -	253.7	0.587				
BMISDS	-5.6	-92.6 -	81.3	0.899				
Motor skill (MABC)^c^	256.9	-178.8 -	692.5	0.248	201.4	-228.9	631.6	0.359
Preschool care^d^	164.1	6.4 -	321.6	0.041*	173.1	19.1	327.2	0.028*
Riskgroup^f^	164.1	-66.8 -	395.1	0.164				
Maternal PA^a^	.163	.022 -	0.305	0.024*	.160	0.020	0.301	0.026*
Paternal PA	.060	-.080 -	0.199	0.401	.045	-0.093	0.184	0.520
Parental education^b^	-171.3	-441.8 -	99.3	0.215	-221.0	-482.1	40.0	0.097
Nordic family^g^	-75.8	-366.3 -	214.6	0.609				
Season^e^	-359.6	-597.9 -	-121.2	0.003*	-370	-607.3	-132.3	0.002*

Linear mixed intercept only models. Child total PA was included as the dependent variable, nested to each individual code.

Parental activity was imputed as a fixed factor as was; parental education (high education as reference), motor skill (high as reference), preschool care (part time as reference), season (summer as reference vs. winter), sex (boy as reference), BMISDS, risk group ..(Low risk as reference) and Nordic family (Nordic as reference).

Model I is a full model including all variables of interest and additional potential confounding factors.

Model II is a forward stepwise selection model with the threshold set to *p*-value <0.3 from bivariate models (Additional file 8) this model also takes model I in consideration by including the significant (*p*-values <0.05) variables from this model. * *p*-value significant at 0.05 level

^a^ PA=physical activity

^b^ Parental education was considered high if at least one parent had an academic education

^c^ Movement ABC test for motor skills High considered >15^th^ percentile

^d^ Full-time preschool care considered ≥30h per week

^e^ Current season for PA measurement, spring (March-May), summer (June-August), fall (September-November) and winter (December-February)

^f^ Risk group classified as obesity risk based on parental BMI Low risk parental BMI <25kg/m high risk parental BMI >25kg/m

^g^ Nordic family was considered if both parents originate from a Nordic country

^h^ Coef.= unstandardized regression coefficient

^i^ CI= confidence interval

Season of year had no interaction with maternal PA nor child time in preschool (data not shown). Paternal PA was significantly positively related to boys PA at age two. Except from that, no significant relation between paternal PA and child PA was found. Maternal PA was significantly positively related over time in the LMM, and cross-sectionally significantly related to both boys’ and girls’ PA. Sex, weight status, motor skills, risk group, SES, and family origin were never significantly related to PA when analyzed longitudinally nor cross sectionally.

## Discussion

This study evaluated longitudinal patterns and correlations of physical activity in children of two to six years of age. We found that accelerometer-measured PA in children increased over time and the daily pattern changed during two to six years of age. From three years of age, the children had an uneven distribution of PA across the week, with higher activity during weekdays. Moreover, a positive significant correlation between child accelerometer-measured PA and maternal accelerometer-measured PA over time was observed. However, we did not find any significant differences in PA between boys and girls, nor could we find that weight status, motor skills or SES was significantly related to PA.

The finding that children´s PA increased over time is in line with recent findings describing a peak in PA at six years of age [8]. Our data complements the PA pattern evolvement as we also provide data from two-year-old children. The finding of an increase in PA by age can have several explanations. During the observed years children experience a rapid motor development that could positively affect their ability to be active and therefore increase the CPM [44, 58]. Furthermore, with age, upper limb movements become straighter and faster [59], which, in combination with increasing arm length may contribute to the increase in accelerometer-measured PA, since the accelerometer was placed on the wrist [8]. The increase in CPM could also potentially be due to increased arm movements. An overall increase in child PA during these years among Swedish children has not been reported. Official numbers among the youngest children are scares. However, among school aged children the time trend shows that the PA has been stable or decreased during the same years as these measurements took place [60].

From three years of age, the children had an uneven distribution of PA across the week, with a higher amount of PA during the weekdays. This could be because Swedish preschools often schedule times for outdoor play [50, 61]; a possibility that our results strengthen, since time in preschool showed a significant correlation to child accelerometer-measured PA (Table 2). However, the magnitude of these differences varied between the least and most active children. The least active children did not differ in activity between weekdays and weekend days.

Parents were more active on weekend days than on weekdays independently of the age of their child. It has been suggested that parents don’t have the same time to be active with their children, or that they consider PA as something the preschools should provide [34, 62]. However, maternal accelerometer-measured PA was significantly related to child accelerometer-measured PA, with a medium to large effect size. This contradicts the assumption that parents do not interact in activity with their children. More likely, the uneven distribution of PA between weekdays and weekend days is explained by the preschools’ capacity for planned out/indoor play, together with others, in safe environments customized for children [62]. Even so, the least active children are also the ones without a difference in PA between weekdays and weekend days. They are, even without a decrease in activity on weekend days, still less active than the most active children during this time. This supports the argument that PA interventions in preschools could be beneficial and underline the importance of a preschool PA curriculum. This has, to our knowledge, not been reported previously and future research should try to characterize this lowest tertile, as it is the most important group to target in interventions aiming to enhance PA.

Over the years, child accelerometer-measured PA increases on an average level and becomes more evenly distributed throughout the day (Fig. 2 and Additional file 4). This is probably because younger children nap during the day [8, 25, 40, 63, 64]; however, peak hour remained around the same time across all years with an increased VM CPM (additional file 2). PA was higher during the summer compared to the winter, which has also been reported previously [41, 65]. Hence, potential seasonal variations need to be taken into consideration when interventions are designed and evaluated.

In our study we could not find differences in PA between boys and girls, neither when analyzed longitudinally nor cross sectionally. Previously, it has been extensively reported that boys are more physically active than girls [6, 9–11]. However, our results are in line with previous studies including children at the same age using accelerometry [8, 66]. The absence of sex differences before 6 years of age may indicate that the differences found in older children are due to social factors rather than differences in biologically induced spontaneous PA between boys and girls. We have previously found that the differences between boys and girls 6-10 years old were significant only during school time which supports this hypothesis [41].

In the present study, maternal accelerometer-measured PA was significantly related to child accelerometer-measured PA from two to six years of age. Previously, parental-child PA relationships have been reported with inconsistent results and the ages of the children are mostly above six years of age [26–38]. We could not find evidence of child sex differences in the correlation between parental and child accelerometer-measured PA, which suggests that the associations were similar for boys and girls, in line with a previous study [34]. During the first years of a child’s life, they spend more time with their parents than later in life, and it is not uncommon that mothers spend more time with their children than fathers [67]. This could possibly explain the differences in reported associations with age and sex, with a stronger association between mothers' and children's PA in preschool age [27, 28, 34]. It is important to acknowledge that the direction of causality is unknown, and it is possible that an active child drives the mothers PA. Nevertheless, parental accelerometer-measured PA is still an interesting factor, especially as it has been demonstrated previously that interventions involving the whole family seem to offer slightly better effectiveness for enhancing child PA [4].

### Strength and limitations

One important strength of this study is the frequent follow-up of PA in children at this young age with a high wear compliance (number of valid days 6.74 of 7). This, together with the accelerometry provide robust data to make conclusions on how child accelerometer-measured PA change over time.

An apparent limitation in the present study is the number of missing cases. Attrition of the study sample is unfortunately common in longitudinal studies ( [68, 69]) and when following families during the toddler years it is expected to lose participants. However, the statistical method was chosen with this limitation in mind and LMM is a flexible tool with the advantage of being able to deal with missing values [55]. We believe that the internal validity is strong, however the external validity and the generalizability of our results might be limited. We performed drop-out analyses and found that excluded families were more likely to be in the high-risk group (*p<.010)* and had lower educational levels (*p=.035)* than included families. It is possible that this could have affected the results in regard to the SES relationship to child accelerometer-measured PA and our findings should be interpreted with the high SES-status in mind. Since data in the present study is collected from an RCT it is important to consider the possibility of an intervention effect affecting the results. However, since we excluded the intervention group and the control and reference group did not receive any additional information, other than information usually provided by the health care centers in Sweden regarding the topics for the RCT, we do not consider a possible intervention effect as an affecting factor for our results.

## Conclusion

PA increased on average with 11% per year, similarly for boys and girls and from three years of age children were more active during weekdays than weekend days. These results indicate that child PA benefits from active stimulation by parents and care takers already from early ages. The findings herein also indicate that children might benefit from different setups of interventions and future studies should focus on identifying attributes of possible intervention designs for weekend days as well as characterize the least active children. Moreover, the seemingly natural increase in PA over these young years needs to be consider when interpreting results from interventions, and not to be assigned to any specific intervention per se.

## Supplementary Information

**Additional file 1.** Portable Document Format, PDF. Flowchart of study population. A visual description of the inclusion of participants in the study.

**Additional file 2.** Portable Document Format, PDF. Hourly pattern on (A) weekdays and (B) weekend days for child physical activity, all years. A figure showing the hourly physical activity patterns for the children for all years, age two to six.

**Additional file 3.** Portable Document Format, PDF. Child physical activity by weekdays – All years. A figure showing the weekly mean of child physical activity for all years, age two to six.

**Additional file 4.** Portable Document Format, PDF. Descriptive characteristics of participants per year. Means and standard deviations of physical activity data as well as number of observations, age, weight status and motor skills for children and parents.

**Additional file 5.** Portable Document Format, PDF. Differences in physical activity on weekend days by tertiles. A table showing differences in physical activity on weekend days between tertiles, including the Bonferroni correction for multiple testing.

**Additional file 6.** Portable Document Format, PDF. Total physical activity differences between mothers and fathers. A table showing differences in physical activity between mothers and fathers at all years.

**Additional file 7.** Portable Document Format, PDF. Hourly pattern of parental activity. A figure showing hourly patterns of parental activity on weekdays and weekend days year 2 and 6.

**Additional file 8.** Portable Document Format, PDF. Correlations over time to child physical activity – Bivariate models. A table showing the results from bivariate models using linear mixed effect models.

## Data Availability

The datasets generated and/or analyzed during the current study are not publicly available. Data can indirectly be traced back to the study participants, and according to Swedish and EU personal data legislation, this means that access can only be made upon request. The request should in this case be addressed to the PI Claude Marcus (claude.marcus@ki.se) and will be handled on a case-by-case basis. Any sharing of data will be regulated via a data transfer and use agreement with the recipient.

## Abbreviations

BMI: Body mass index
BMISDS: Body mass index standard deviation score
CPM: Counts per minute
Early STOPP: Early STockholm obesity prevention project
IOTF: International obesity task force
LMM: Linear mixed effect model
MABC-2: Movement assessment battery for children 2^nd^ edition
PA: Physical activity
SES: Socioeconomic status
VM: Vector magnitude
WHO: World Health Organization
