# Supplementary figures and images for: Physical activity in early childhood: a five-year longitudinal analysis of patterns and correlates
Source: Int J Behav Nutr Phys Act. 2022 Apr 20;19:47. doi: 10.1186/s12966-022-01289-x (PMC9022334; doi:10.1186/s12966-022-01289-x)

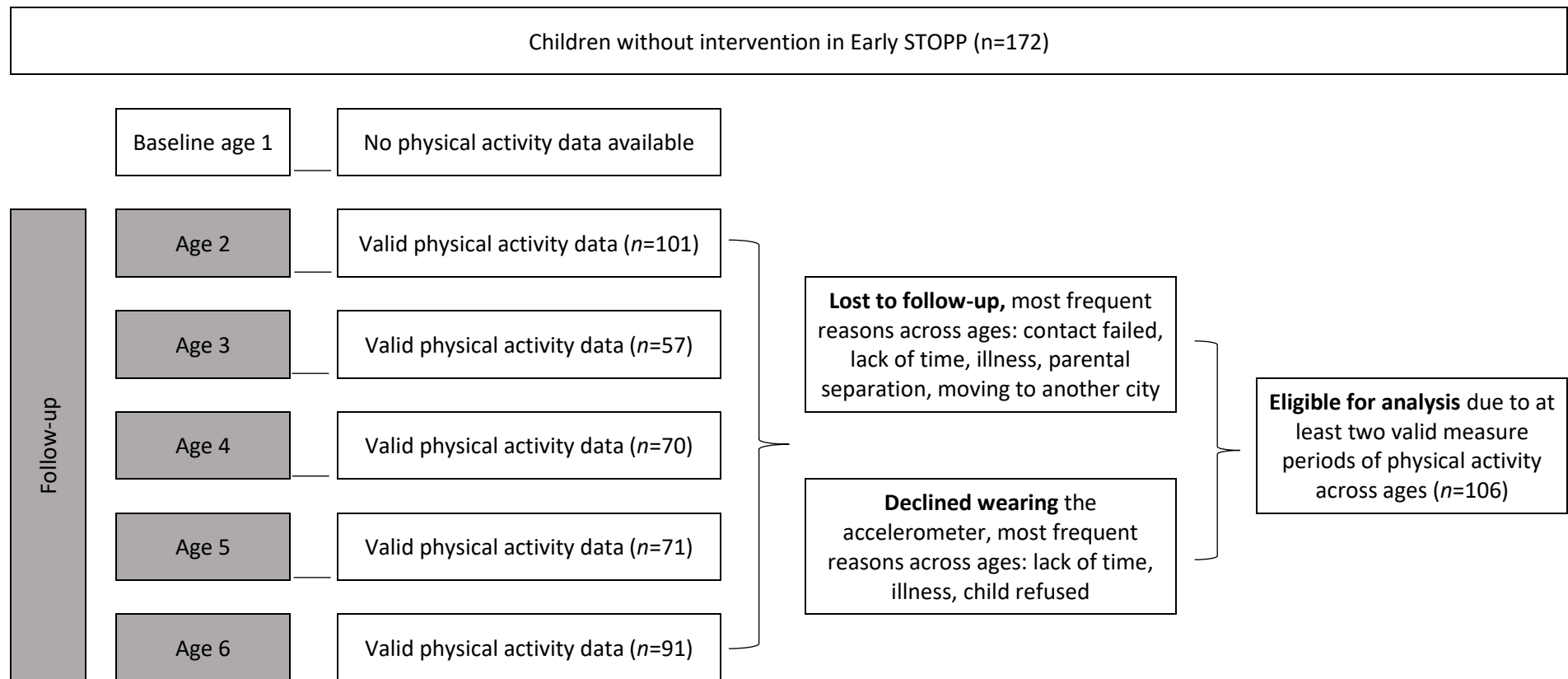

**Additional file 1.** Flowchart of study population

Supplement: Supplementary file 1 — Additional file 1. Portable Document Format, PDF. Flowchart of study population. A visual description of the inclusion of participants in the study. [file 12966_2022_1289_MOESM1_ESM.pdf]

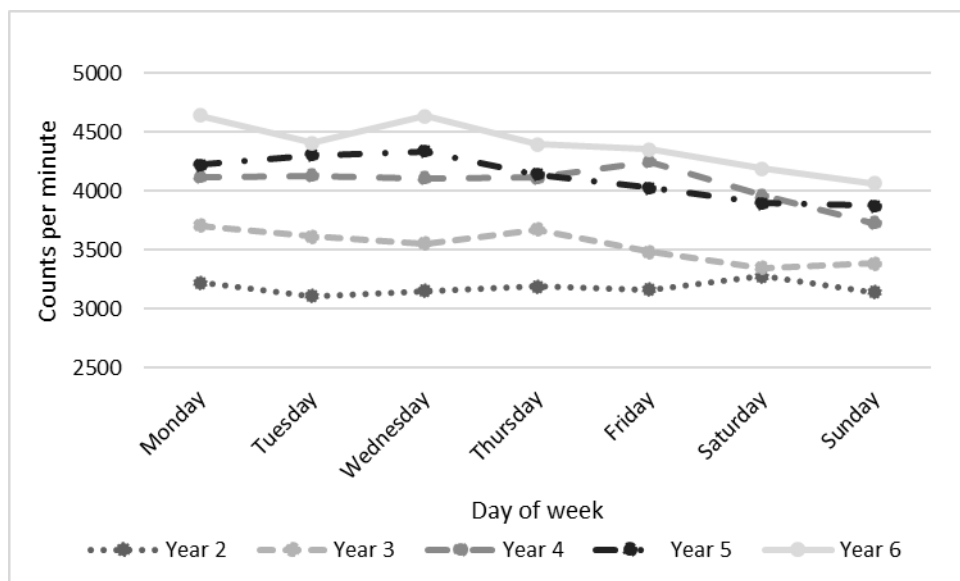

**Additional file 3.** Child physical activity by weekdays – All years

Supplement: Supplementary file 3 — Additional file 3. Portable Document Format, PDF. Child physical activity by weekdays – All years. A figure showing the weekly mean of child physical activity for all years, age two to six. [file 12966_2022_1289_MOESM3_ESM.pdf]
