# Supplementary material for: Physical activity in early childhood: a five-year longitudinal analysis of patterns and correlates
Source: Int J Behav Nutr Phys Act. 2022 Apr 20;19:47. doi: 10.1186/s12966-022-01289-x (PMC9022334; doi:10.1186/s12966-022-01289-x)
Supplement: Supplementary file 2 — Additional file 2. Portable Document Format, PDF. Hourly pattern on (A) weekdays and (B) weekend days for child physical activity, all years. A figure showing the hourly physical activity patterns for the children for all years, age two to six. [file 12966_2022_1289_MOESM2_ESM.pdf]

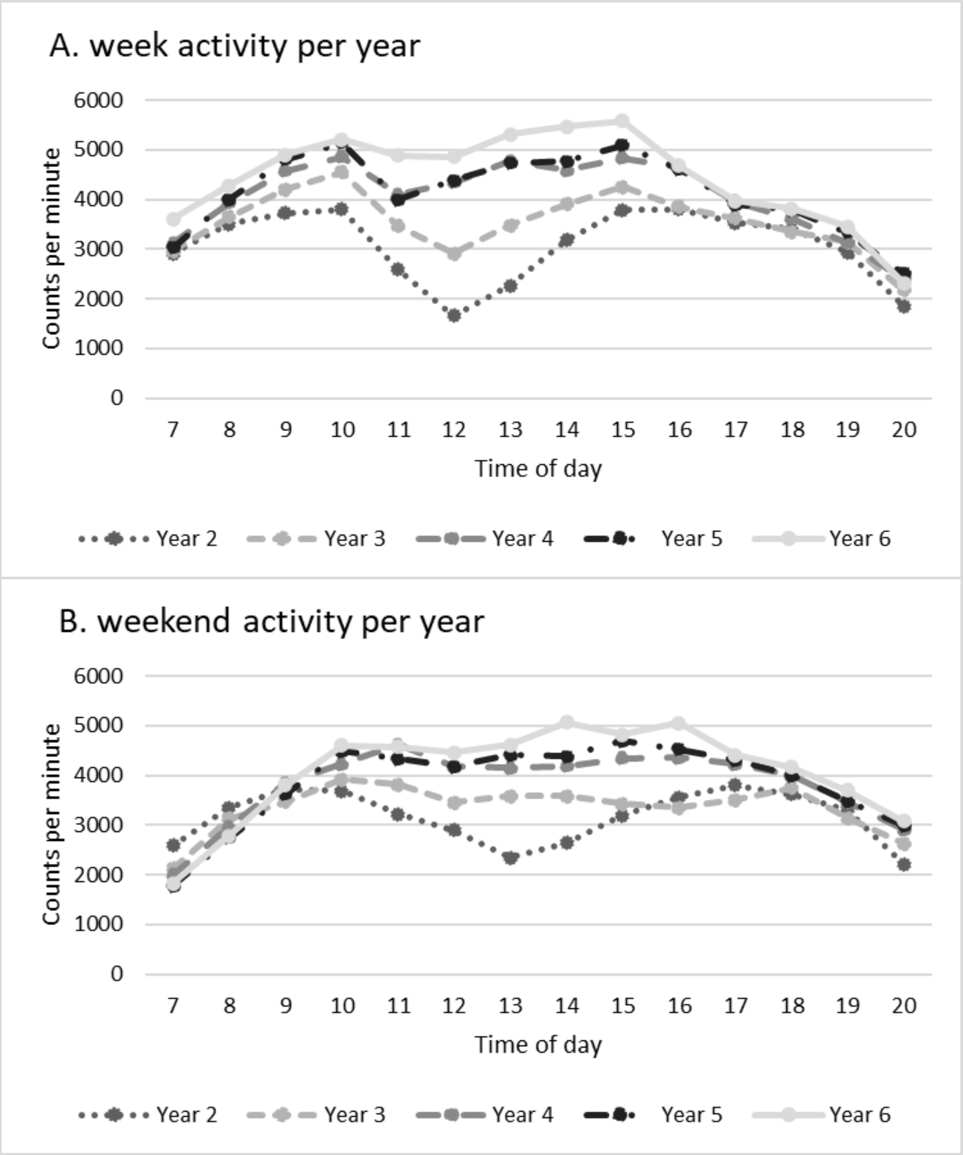

Additional file 2. Hourly pattern on (A) weekdays and (B) weekend days for child physical activity, all years.
