# Supplementary material for: Physical activity in early childhood: a five-year longitudinal analysis of patterns and correlates
Source: Int J Behav Nutr Phys Act. 2022 Apr 20;19:47. doi: 10.1186/s12966-022-01289-x (PMC9022334; doi:10.1186/s12966-022-01289-x)
Supplement: Supplementary file 4 — Additional file 4. Portable Document Format, PDF. Descriptive characteristics of participants per year. Means and standard deviations of physical activity data as well as number of observations, age, weight status and motor skills for children and parents. [file 12966_2022_1289_MOESM4_ESM.pdf]

Additional file 4. Descriptive characteristics of participants per year

Year	2	3	4	5	6
Child Number of observations	86	56	70	70	87
Child age mean (SD)	2.04 (0.07)	3.07 (0.09)	4.03 (0.07)	5.03 (0.6)	6.09 (0.26)
Child CPM Week mean (SD)	3175 (521)	3602 (619)	4137 (733)	4199 (530)	4503 (805)
Child CPM Weekend mean (SD)	3210 (624)	3364 (702)	3870 (762)	3891 (764)	4125 (848)
Child CPM Total mean (SD)	3188 (523)	3541 (571)	4056 (666)	4107 (534)	4392 (733)
Child number of valid days mean (SD)	6.8 (0.52)	6.7 (0.54)	6.6 (0.75)	6.7 (0.54)	6.9 (0.35)
Child BMISDS[a] mean (SD)	-0.08 (1.08)	0.09 (0.93)	0.23 (0.95)	0.41 (1.01)	0.15 (1.1)
Child weight status[b] % normal weight	81	83	82	78	86
Child motor skills[c] mean (SD)					76.84 (12.78)
Child motor skills cut off % of children with sufficient[d] motor skills					82
Mother Number of observations	89	62	73	74	83
Mother CPM Week mean (SD)	2649 (561)	2574 (603)	2481 (578)	2444 (456)	2511 (569)
Mother CPM Weekend mean (SD)	2703 (634)	2630 (525)	2633 (654)	2645 (502)	2647 (620)
Mother CPM Total mean (SD)	2666 (548)	2591 (521)	2526 (537)	2504 (336)	2547 (523)
Mother number of valid days mean (SD)	6.9 (0.49)	6.7 (0.66)	6.8 (0.53)	6.9 (0.28)	6.9 (0.47)
Father Number of observations	87	61	76	70	85
Father CPM Week mean (SD)	2267 (643)	2111 (546)	2196 (592)	2255 (597)	2099 (535)
Father CPM Weekend mean (SD)	2391 (601)	2220 (575)	2260 (611)	2369 (617)	2255 (567)
Father CPM Total mean (SD)	2305 (580)	2144 (497)	2215 (514)	2296 (538)	2143 (477)
Father number of valid days mean (SD)	6.8 (0.44)	6.9 (0.47)	6.8 (0.52)	6.8 (0.5)	6.9 (0.28)

Abbreviations: SD=standard deviation, CPM=counts per minute, BMISDS=Body mass index standard deviation score

[a] BMISDS based on the international reference provided by the International Obesity Task Force (IOTF)

[b] Weight status based on the international reference provided by the International Obesity Task Force (IOTF)

[c] Child motor skills evaluated based on movement ABC 2nd edition

[d] Sufficient motor skills - children that scored above the 15th percentile
