# Supplementary material for: Physical activity in early childhood: a five-year longitudinal analysis of patterns and correlates
Source: Int J Behav Nutr Phys Act. 2022 Apr 20;19:47. doi: 10.1186/s12966-022-01289-x (PMC9022334; doi:10.1186/s12966-022-01289-x)
Supplement: Supplementary file 5 — Additional file 5. Portable Document Format, PDF. Differences in physical activity on weekend days by tertiles. A table showing differences in physical activity on weekend days between tertiles, including the Bonferroni correction for multiple testing. [file 12966_2022_1289_MOESM5_ESM.pdf]

Additional file 5. Differences in physical activity on weekend days by tertiles.

Year	Activity group[a]	N	Mean PA weekend days	p-value for model[b]
2	Low	28	2723	<0.001*
	Medium	29	3105	
	High	29	3786	
3	Low	18	3072	<0.001*
	Medium	19	3171	
	High	19	3835	
4	Low	23	3404	<0.001*
	Medium	24	3790	
	High	23	4418	
5	Low	23	3403	<0.001*
	Medium	24	4026	
	High	23	4244	
6	Low	29	3710	<0.001*
	Medium	29	4077	
	High	29	4589	

* p-value significant at 0.05 level

[a] Activity group based on tertiles

[b] p-value for the full model. Adjustment for multiple comparison using Bonferroni reveals that:
at year 2 low vs. medium p=0.005, low vs. high p=<0.001, medium vs. high p=<0.001
at year 2 low vs. medium p=1.0, low vs. high p=<0.001, medium vs. high p=0.006
at year 4 low vs. medium p=0.135, low vs. high P=<0.001, medium vs. high p=0.004
at year 5 low vs. medium p=0.009, low vs. high <0.001, medium vs. high p=0.848
at year 6 low vs. medium p=0.224, low vs. high <0.001, medium vs. high p=0.042
