# Supplementary material for: Physical activity in early childhood: a five-year longitudinal analysis of patterns and correlates
Source: Int J Behav Nutr Phys Act. 2022 Apr 20;19:47. doi: 10.1186/s12966-022-01289-x (PMC9022334; doi:10.1186/s12966-022-01289-x)
Supplement: Supplementary file 6 — Additional file 6. Portable Document Format, PDF. Total physical activity differences between mothers and fathers. A table showing differences in physical activity between mothers and fathers at all years. [file 12966_2022_1289_MOESM6_ESM.pdf]

Additional file 6. Total physical activity differences between mothers and fathers

	Mother CPM[a] (SD[b])	Father CPM (SD)	*P-value*
Age 2	2649 (561)	2267 (643)	<0.001*
Age 3	2574 (603)	2111 (546)	<0.001*
Age 4	2481 (578)	2196 (592)	0.004*
Age 5	2444 (456)	2258 (597)	0.037*
Age 6	2511 (569)	2099 (535)	<0.001*

*P value significant at 0.05 level

[a] CPM = Counts per minute

[b] SD = Standard deviation
