# Supplementary material for: Physical activity in early childhood: a five-year longitudinal analysis of patterns and correlates
Source: Int J Behav Nutr Phys Act. 2022 Apr 20;19:47. doi: 10.1186/s12966-022-01289-x (PMC9022334; doi:10.1186/s12966-022-01289-x)
Supplement: Supplementary file 7 — Additional file 7. Portable Document Format, PDF. Hourly pattern of parental activity. A figure showing hourly patterns of parental activity on weekdays and weekend days year 2 and 6. [file 12966_2022_1289_MOESM7_ESM.pdf]

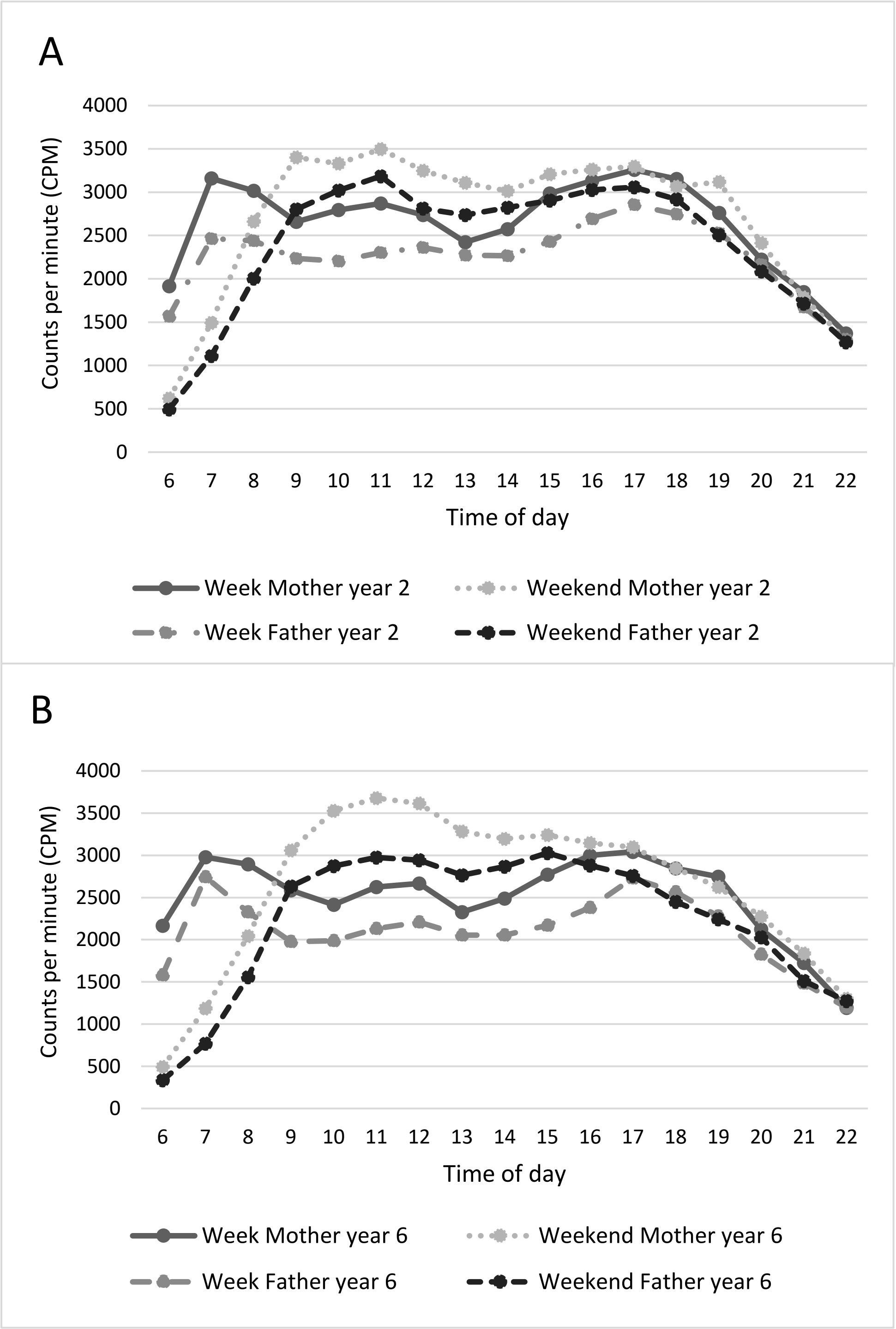

Additional file 7. Hourly pattern of parental activity

7A shows hourly pattern of parental physical activity on weekdays and weekend days separately at child age two

7B shows hourly pattern of parental physical activity on weekdays and weekend days separately at child age six
