# Supplementary material for: Physical activity in early childhood: a five-year longitudinal analysis of patterns and correlates
Source: Int J Behav Nutr Phys Act. 2022 Apr 20;19:47. doi: 10.1186/s12966-022-01289-x (PMC9022334; doi:10.1186/s12966-022-01289-x)
Supplement: Supplementary file 8 — Additional file 8. Portable Document Format, PDF. Correlations over time to child physical activity – Bivariate models. A table showing the results from bivariate models using linear mixed effect models. [file 12966_2022_1289_MOESM8_ESM.pdf]

Additional file 8. Correlations over time to child physical activity - Bivariate models

	Coefficient	95% CI	p-value
Maternal PA	0.156	0.028 – 0.283	0.016*
Paternal PA	0.070	-0.057 – 0.196	0.282
BMI SDS	25.2	-47.8 – 98.1	0.499
Weight status[a]	38.5	-553.2 – 630.1	0.899
Sex	130.4	-290.4 – 551.1	0.544
Risk group[b]	-30.0	-830.2 – 770.2	0.941
Parental education[c]	-661.5	-1174.4 – -148.6	0.011*
Motor skill (MABC)[e]	325.8	-273.2 – 924.8	0.286
Nordic family[h]	61.0	-199.3 – 321.4	0.643
Summer season[f]	-102.6	-700.8 – 495.5	0.737
Part-time preschool care[g]	90.2	-56.0 – 236.5	0.226

Intercept only models. Here presented in one table but each factor was analyzed separately.

Child total PA was included as the dependent variable, nested to each individual code. Parental activity was inputted as a fixed factor as was; BMISDS, weight status (normal weight as reference), sex (boy as reference), risk group (Low risk as reference), parental education (high education as reference), motor skill (Low as reference), Nordic family (Non-Nordic as reference), season (summer as reference vs. winter) and preschool care (part time as reference).

* p-value significant at 0.05 level

[a] Normal weight or overweight/obese based on international cut offs by Cole et al

[b] Risk group classified as obesity risk based on parental BMI Low risk parental BMI <25kg/m high risk parental BMI >25kg/m

[c] Parental education was considered high if at least one parent had an academic education

[d] The Hempel test for motor skill, NOS 0-58, high considered >53

[e] Movement ABC test for motor skills High considered >15th percentile

[f] Current season for PA measurement, spring (March-May), summer (June-August), fall (September-November) and winter (December-February)

[g] Full-time preschool care considered ≥30h per week

[h] Nordic family was considered if both parents originate from a Nordic country
